# Long transportation duration affects nutrient composition, mycotoxins and microbial community in whole-plant corn silage

**DOI:** 10.3389/fvets.2023.1189358

**Published:** 2023-05-18

**Authors:** Caixia Zhang, Jun Jiang, Junfeng Li, Jiming Zhang, Xinyue Zhang, Hairong Wang

**Affiliations:** ^1^College of Animal Science and Technology, Inner Mongolia Agricultural University, Hohhot, China; ^2^Inner Mongolia Yili Industrial Group Co., Ltd., Hohhot, China; ^3^National Center of Technology Innovation for Dairy, Hohhot, China

**Keywords:** corn silage, nutritional quality, mycotoxin, microorganism, transport time

## Abstract

**Introduction:**

Potential nutrient losses and mycotoxin accumulation caused by abnormal fermentation during transportation from cropland to dairy farms leads to the diseases incidence and threatens the health of dairy cows, then further causes financial losses. The aim of this study was to investigate the effects of different transportation times on the nutritional composition, mycotoxins, and microbial communities in whole-plant corn silage (WPCS).

**Methods:**

Three groups were subjected to different transport times: DY, short (<200 min); ZY, medium time (300–500 min); and CY, long transport time (>600 min). WPCS were collected from the same field, and nutrient composition and microbial composition before and after transportation were analyzed.

**Results and discussion:**

Our results showed that the temperature of WPCS was higher in the ZY and CY groups than in the DY group (*P* < 0.01). There were no significant differences in dry matter (DM), crude protein (CP), neutral detergent fiber (NDF), acid detergent fiber (ADF), ether extract (EE) and starch contents after different transportation times (*P* > 0.05), whereas the starch and water-soluble carbohydrates (WSC) contents in the CY group was significantly decreased after transport (*P* < 0.05). Similarly, the concentration of vomitoxin in the DY and CY groups declined markedly (*P* < 0.05) and the zearalenone content in the DY group also significantly decreased after transportation (*P* < 0.05). Regarding the analysis of microorganisms in WPCS, UniFrac-distance matrices and Shannon indices showed differences in the ZY group (*P* < 0.05), but fungal diversities were not influenced by the transport time (*P* > 0.05). In the ZY group, the relative abundance of *Lactiplantibacillus* decreased significantly after transportation (*P* > 0.05), but the relative abundances of *unidentified*_*Chloroplast, Pantoea, Gluconobacter, unidentified Acetobacter* and *Acinetobacter* increased markedly (*P* < 0.05). In addition, the relative abundances of *Acetobacter* and *Gluconobacter* in the CY group increased after transport (*P* < 0.05). Among fungal communities, a total of three, nine, and ten different fungal flora were observed in the DY, ZY, and CY groups, respectively, although no difference was found in fungal diversity. In conclusion, increased temperature, loss of starch, and mycotoxin variation were found with increased transport time. This might be the result of competition between bacteria and fungi, and novel technologies will need to be utilized for further exploration of the mechanism.

## 1. Introduction

Currently, China is the third largest dairy producer in the world (1). Over the past century, tremendous improvements have been witnessed in Chinese dairy farms, and this rapid growth has been accompanied by fundamental changes in production systems, from traditional modes to intensive systems. Correspondingly, the sustainable supplementation of feedstuffs such as whole-plant corn silage (WPCS) has become critically important for this fast-growing dairy industry.

As the most popular source of roughage, WPCS accounts for more than 40% of the total diet in dairy farming (2). Many advantages have contributed to the high inclusion of WPC by dairy farmers, including lower harvesting costs, minimized risks of production, elevated yield, and flexible harvest (3). More importantly, in comparison with other forages, WPCS provides uniquely high energy (mainly from starch in the kernel fraction) and physically effective neutral detergent fiber (NDF, peNDF) concurrently (3, 4). Corn silage is the most common forage in the United States, and its production has increased by more than 30% since the 1980's (5).

Ensiling is a common approach for conserving feedstuffs. Approximately 150,000 tons of silage were produced around the world every year (6). In general, high-quality silage is produced by anaerobic fermentation technology. It dominated by lactic acid bacteria (LAB), which produce lactic acid to decrease pH and inhibit the undesirable microorganism (6). However, decreased nutritional quality of forage and potential health risks to animals induced by aerobic bacteria may be discovered after transportation from the field to the destination. A previous study suggested that exposure of chopped roughage to the atmosphere contributed to increased feed temperature and pH because of the growth and multiplication of yeast, mold, as well some aerobic bacteria (7). Hu et al. (8) showed that the relative abundance of *Lactobacillus* increased sharply when WPCS was exposed to oxygen for ~72 h. In addition, fungi in silage may produce corresponding toxins with abnormal fermentation, and contamination with mycotoxins can endanger the performance and health of livestock and human beings (9, 10). However, limited information has been systematically observed on the microbial variation of WPCS in the process of transportation, and studies related to the fermentation suppression of growth of harmful bacteria would provide new insights for the exploration of silage additives.

The present research aimed to investigate the effects of abnormal fermentation caused by long transportation times on the nutritional quality, mycotoxin content, and microbial community of WPCS. We hypothesized that long transportation time would contribute to nutrient loss and mycotoxin growth by altering microbial diversity and composition.

## 2. Materials and methods

### 2.1. Sample collection

WPCS was harvested from the same region (42° 01′ 17.83 “N, 121° 40′ 13.17” E; Tongliao, China) at about the wax ripening period. The samples were harvest and directly chopped into 1–2 cm (CLASS JAGUAR-800, Beijing Debon Dawei Technology Co., Ltd, Beijing, China), then loaded into tractor trailer (length: 7.2 m, width: 3.4 m, height: 3.6 m) with plastic protective film, which ensure the materials in a closed state to prevent pollution caused by external weather during the whole transportation.

The experimental samples were collected before transportation and after arriving at the destination. The silage sampler tool was inserted vertically into the lower third (~70–100 cm from the top of the sampler) of the corn silage, and a total of four points were detected. All samples from four points were mixed and divided into two parts; one was placed at −20°C for the measurement of nutrient and mycotoxin content and the other was stored at −80°C for the analysis of microbial composition.

Three transport times were selected: short (DY, t < 200 min), medium (ZY, 300 min < t < 500 min), and long transport time group (CY, t > 600 min). The total time taken in each group was 140, 390, and 610 min, respectively.

### 2.2. Measurement and analysis

The sample temperature was measured twice (at the beginning and end of transport) at six different corn silage points, at strictly 1 m depth, using a resistance thermometer PDS-100 (Saisidun Technology Co., LTD, Beijing, China). The experimental samples were analyzed according to AOAC (2016) (11) for dry matter (DM, method 950.15), crude protein (CP, method 984.13), ether extract (EE, method 920.39), starch (method 996.11), and ash (method 942.05). NDF and acid detergent fiber (ADF) were determined using the Ankom Fiber Analyzer System (Ankom Technology, Macedon, NY, USA), as described by Van Soest (12), and water-soluble carbohydrates (WSC) was measured according to Mcdonald et al. (13). The concentrations of vomitoxin (deoxynivalenol, DON) and zearalenone (F-2) were estimated using an ELISA rapid test kit (Huaan Magnech Bio-tech Co., Ltd., Beijing, China).

Total DNA of WPCS was extracted using the Phusion^®^ High-Fidelity PCR Master Mix with a GC Buffer kit according to the manufacturer's protocol. The DNA extract was analyzed on a 1% agarose gel, and the concentration was quantified using a NanoDrop 2000 UV–vis spectrophotometer (Thermo Fisher Scientific, Wilmington, DE, USA). The hypervariable region V3-V4 of the 16S rRNA gene was amplified with primer pairs 515F (GTGCCAGCMGCCGCGGTAA) and 806R (GGACTACHVGGGTWTCTAAT) (14) using an ABI GeneAmp^®^ 9700 PCR thermocycler (ABI, Foster City, CA, USA), and primers ITS5–1737F (GGAAGTAAAAGTCGTAACAAGG) and ITS2-2043R (GCTGCGTTCTTCATCGATGC) (15) were used to amplify the fungal ITS1 region. PCR amplification, amplicon purification and quantification, and amplicon sequencing were performed according to the method described by Yi et al. (16). PCR products were extracted from a 2% agarose gel, purified using a TruSeq^®^ DNA PCR-Free Sample Preparation Kit (Axygen Biosciences, Union City, CA, USA) according to the manufacturer's instructions, and quantified using a Quantus™ Fluorometer (Promega, Madison, WI, USA). Purified amplicons were pooled in equimolar amounts and paired-end sequenced on an Illumina NovaSeq 6000 platform (Illumina Inc., San Diego, CA, USA).

The raw 16S rRNA gene sequencing reads were demultiplexed, quality-filtered using Fastp (Version 0.20.1, Haplox, Shenzhen, China) (17), and merged using FLASH version 1.2.7 (The Center for Computational Biology at Johns Hopkins University, MD, USA) (18) with the criteria set according to Kong et al. (19). Operational taxonomic units (OTUs) with a 97% similarity cut-off were clustered using UPARSE version 7.1 (Independent Investigator, CA, USA) (20), and chimeric sequences were identified and removed. The taxonomy of each OTU representative sequence was analyzed using the Mothur Classifier (version v.1.22.2) (21) against the 16S rRNA database (e.g., Silva v138), using a confidence threshold of 0.99 (22). Alpha diversity indices (observed species, Shannon, Simpson) and Good's coverage index were obtained using the alpha rarefaction script in QIIME (23, 24). Principal coordinate analysis (PCoA) was conducted using ggplot2 in R (version 4.0.2; R Foundation for Statistical Computing, Vienna, Austria). An analysis of similarities (ANOSIM) using UniFrac distance matrices was performed to test the statistical differences among the observed microbial profiles. All sequences were deposited in the NCBI Sequence Read Archive (accession number: SRR23871808 to SRR23871831 for 16S rRNA; SRR23872051 to SRR23872074 for fungal ITS1).

### 2.3. Statistical analysis

The original data were organized in Excel 2021. The indicators of WPCS temperature, nutrient content, and mycotoxins were analyzed using the ANOVA model in SPSS (version 19.0; IBM, Armonk, NY, USA). The data were screened for normality using a univariate procedure before analysis. The results are presented as least-squares means and separated using the PDIFF statement when the fixed effect was significant. All results are expressed as mean ± standard error. Differences in alpha diversity indices were analyzed using the Mann-Whitney U test (25), and principal coordinates analysis (PCoA) combined with non-parametric multivariate variance (PERMANOVA) was analyzed based on the UniFrac distance matrix (26). The linear discriminant analysis (LDA) effect size (LEfSe) method was used to determine features with a significant differential abundance of fungi. Cut-off values ≥ 2 and < 0.5 were used for LDA score and *P*-value, respectively.

Spearman's correlation test was used to assess the relationship between selected microbial genera and temperature, nutrient contents, and mycotoxin concentration. The analysis was performed using the SPSS software and plotted using Prism 9 (GraphPad Software, San Diego, CA, USA). Statistical significance was declared at *P* ≤ 0.05, and trends at 0.05 < *P* ≤ 0.10.

## 3. Results

### 3.1. Effects of different transport times on temperature, nutrient and mycotoxin content of WPCS

**Figure 1:**
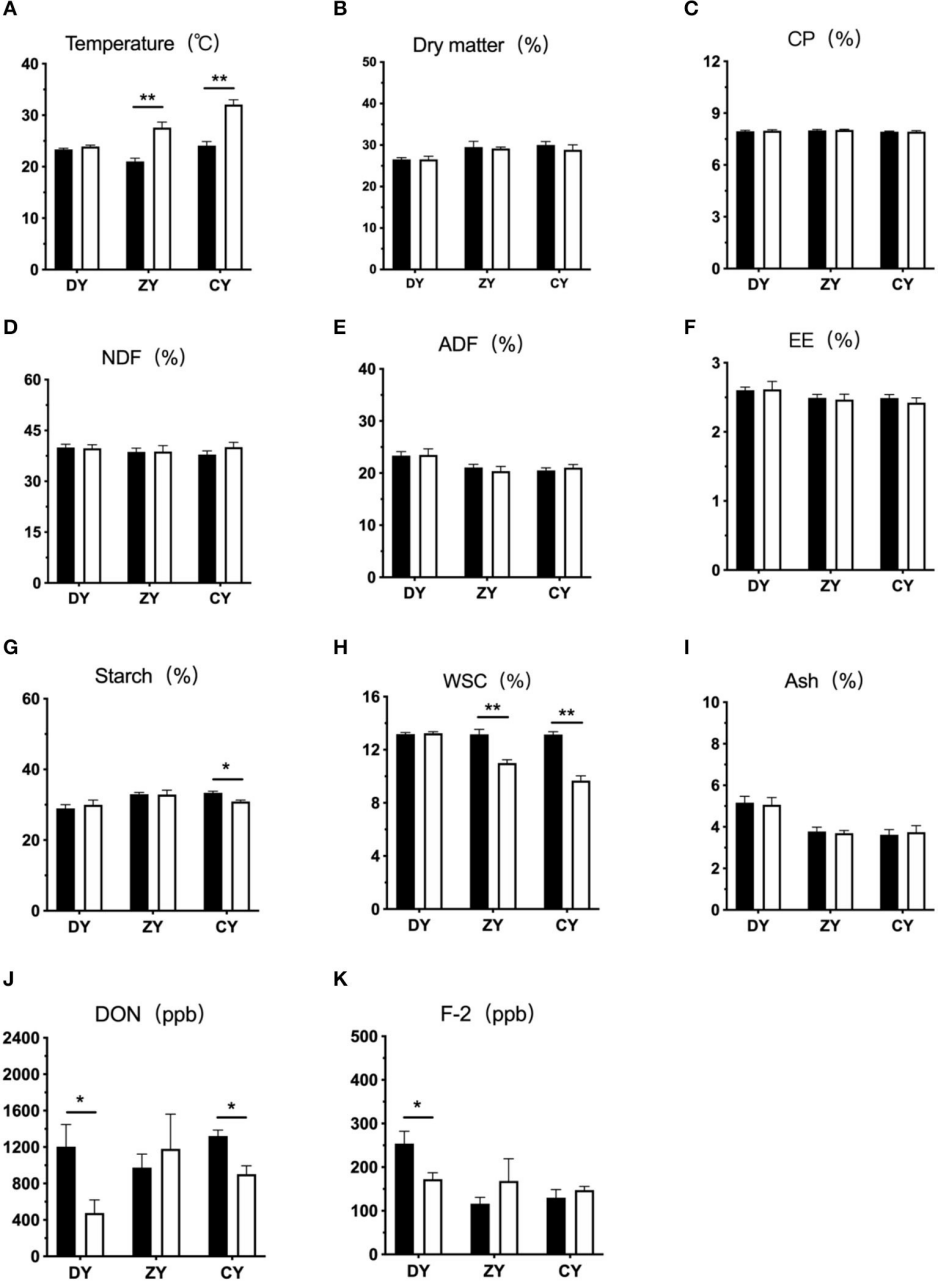
Effects of transport time on phenotypic indicators of whole-plant corn silage. **(A)** temperature, **(B)** dry matter, **(C)** CP, **(D)** neutral detergent fiber, NDF, **(E)** acid detergent fiber, ADF, **(F)** ether extract, EE, **(G)** starch, **(H)** water-soluble carbohydrates, WSC, **(I)** ash, **(J)** vomitoxin, DON and **(K)** zearalenone, F-2. DY, short (*t* < 200 min); ZY, medium (300 min < *t* < 500 min); and CY, long transport time group (*t* > 600 min). ppb: parts per billion. ^*^P < 0.05, ^**^P < 0.01.

As shown in Figure 1, the silage temperature in DY group had no significant change after arriving at dairy farms (*P* > 0.05), but that in the ZY and CY groups was significantly increased (*P* < 0.01). The contents of DM, CP, NDF, ADF, EE, and ash were not influenced by transport time (*P* > 0.05), whereas the starch concentration in the CY group was significantly decreased (*P* < 0.05), the WCS content was also markedly decreased in ZY and CY groups (*P* < 0.05). As for mycotoxin estimation, both DON and F-2 content of WPCS in the DY group were significantly reduced after transportation (*P* < 0.05), and the DON content in the CY group also decreased significantly (*P* < 0.05).

### 3.2. Effects of different transport times on microbial diversity of WPCS

**Figure 2:**
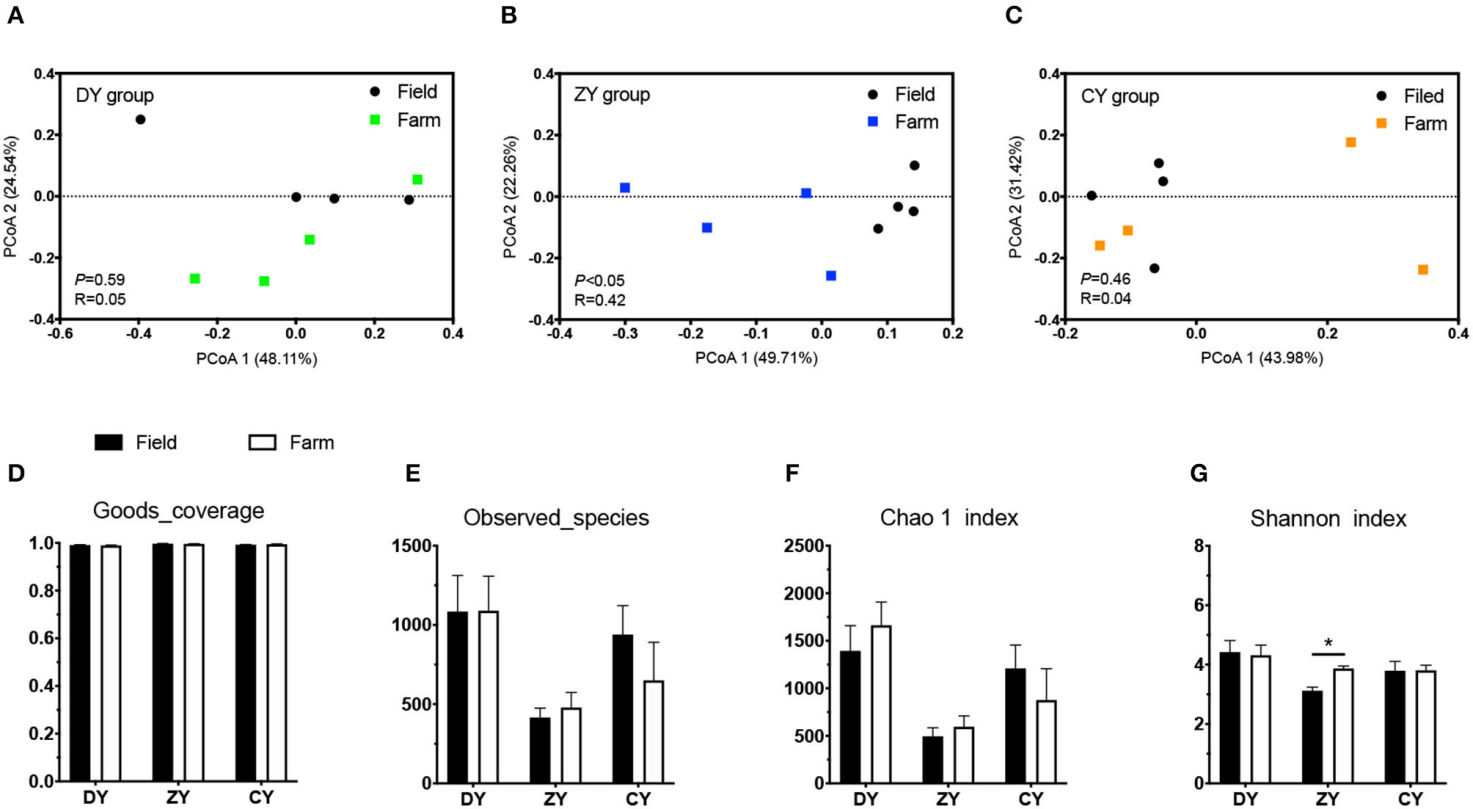
Effects of transport time on bacterial diversity of whole-plant corn silage. Bacterial UniFrac distance matrix analysis of **(A)** short, **(B)** medium, and **(C)** long transport time. **(D)** Sequencing coverage of each group, **(E)** number of species observed, **(F)** Chao 1 index, **(G)** Shannon index. DY, short (*t* < 200 min); ZY, medium (300 min < *t* < 500 min); and CY, long transport time group (*t* > 600 min). ^*^P < 0.05.

**Figure 3:**
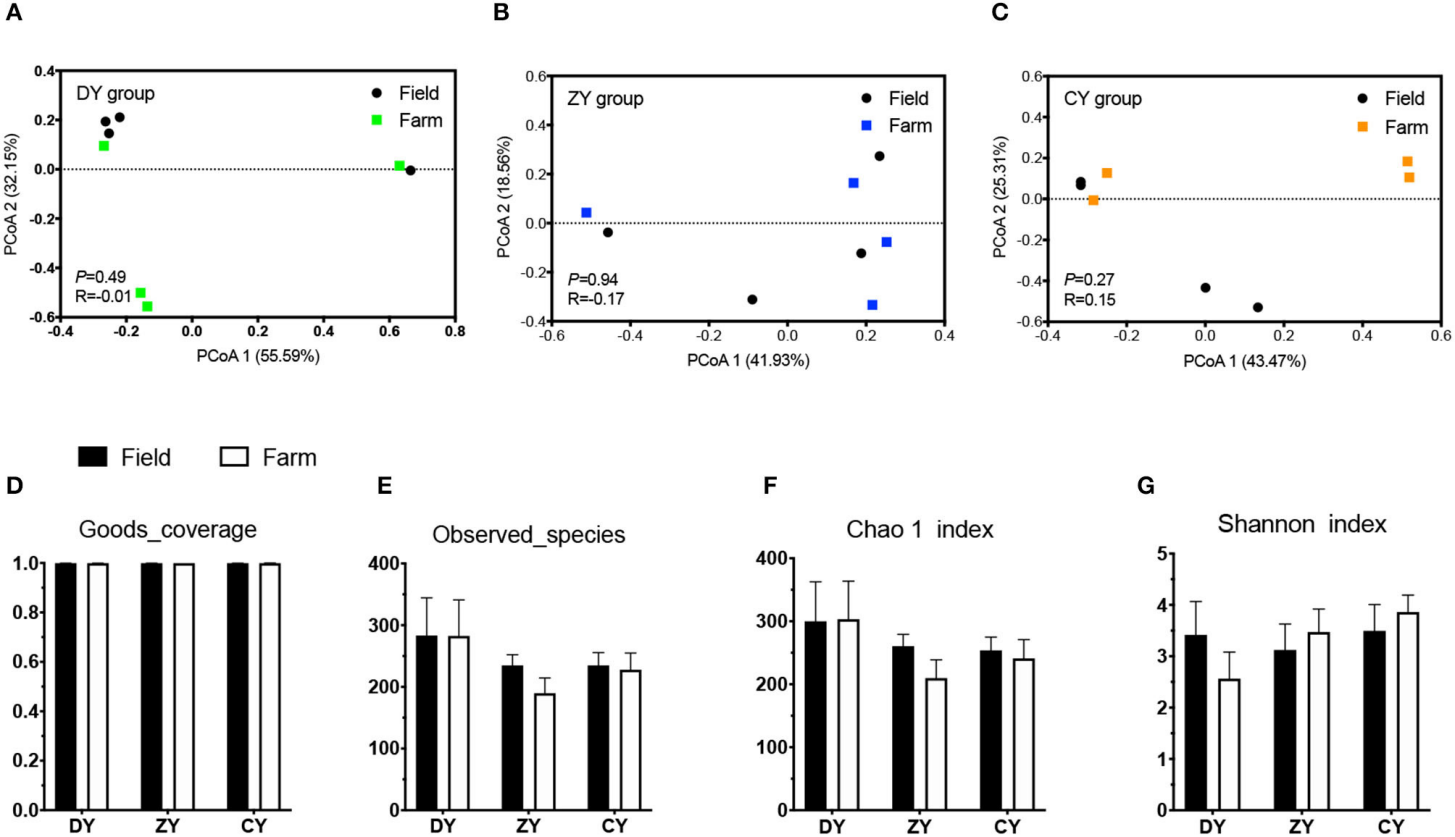
Effects of transport time on fungal diversity of whole-plant corn silage. Fungal UniFrac distance matrix analysis of **(A)** short, **(B)** medium, and **(C)** long transport time. **(D)** Sequencing coverage of each group, **(E)** The umber of species observed, **(F)** Chao 1 index, **(G)** Shannon index. DY, short (*t* < 200 min); ZY, medium (300 min < *t* < 500 min); and CY, long transport time group (*t* > 600 min).

In terms of bacterial diversity, the PCoA plot based on UniFrac distance matrices showed distinct clustering in the ZY group (Figure 2, *P* < 0.05), but not in the DY and CY groups (*P* > 0.05). No significant difference was observed in alpha diversity based on observed_species and Chao1 indices before and after transportation among all three groups (*P* > 0.05); however, the Shannon index increased in the ZY group (*P* < 0.05). The analysis of fungal beta diversity based on UniFrac distance matrices showed no significant differences among all three treatments (Figure 3, *P* > 0.05), the same result was also found for alpha diversity (*P* > 0.05).

### 3.3. Effects of different transport times on microbial taxonomic compositions of WPCS

**Figure 4:**
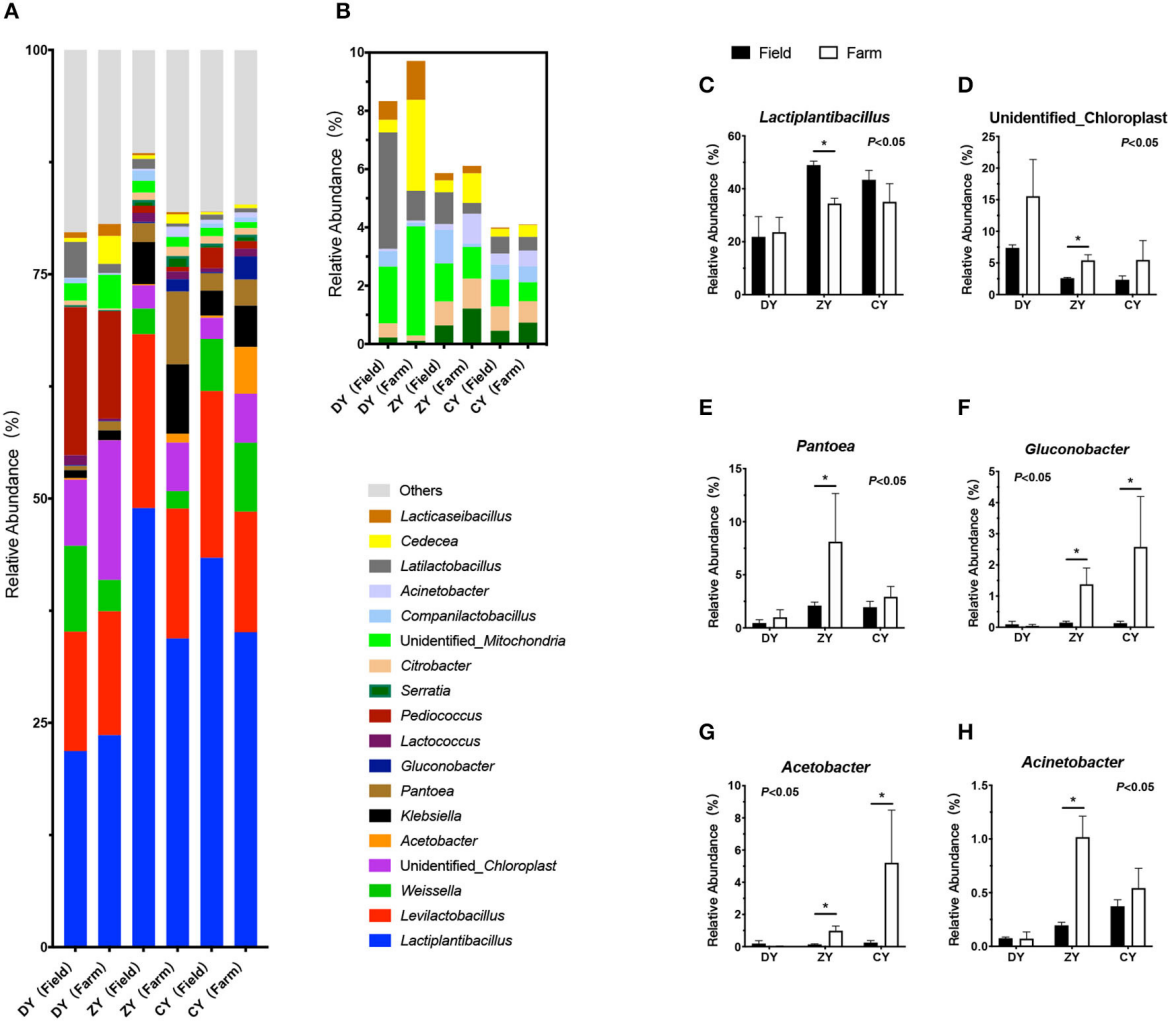
Effects of transport time on bacterial composition of whole-plant corn silage. **(A)** High relative abundance bacterial genera (at least one group of relative abundance >1%). **(B)** Low relative abundance bacterial genera. **(C)** *Lactiplantibacillus*, **(D)** *unidentified_Chloroplast*, **(E)** *Pantoea*, **(F)** *Gluconobacter*, **(G)** *Acetobacter*, **(H)** *Acinetobacter*. ^*^P < 0.05.

As shown in Figure 4, the dominant genera of bacteria in the three groups were *Lactobacillus, Levilactobacillus*, and *Weissella*. The bacterial composition did not change in the DY group (*P* > 0.05). In the ZY group, the relative abundance of *Lactobacillus* decreased significantly after transportation (*P* < 0.05), whereas the relative abundance of *unidentified_Chloroplast, Pantoea, Gluconobacter, Acetobacter*, and *Acinetobacter* increased markedly (*P* < 0.05). In the CY group, the relative abundances of *Gluconobacter* and *Acetobacter* significantly increased after transportation (*P* < 0.05).

**Figure 5:**
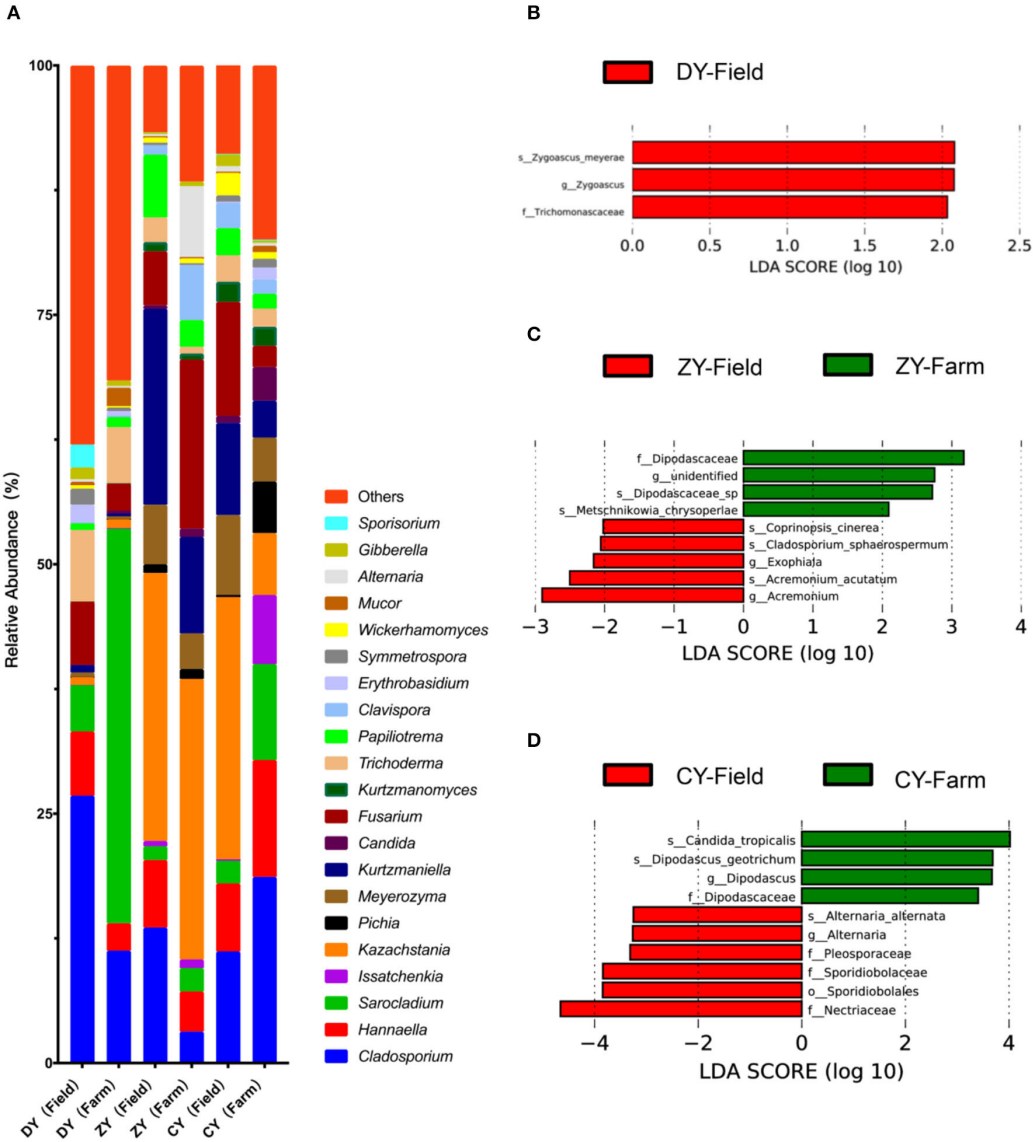
Effects of transport time on fungal composition of whole-plant corn silage **(A)** Relative abundance of fungal genera (at least one group of relative abundance > 1%). LEfSe analysis for fungal colony markers of WPCS in **(B)** DY group, **(C)** ZY group and **(D)** CY group.

The dominant fungal genera *Cladosporium, Sarocladium* and *Kazakhstania*, are shown in Figure 5. LEfSe analysis was used to explore the fungal biomarkers of WPCS transported for different times. The results showed three, nine and ten different fungi in DY, ZY and CY, respectively. Specifically, the relative abundance of fungi *f_Trichomonascaceae, g_Zygoascus, s_ Zygoascus_meyerae* in corn silage harvested from the field disappeared when they were transported to the destination within 200 min (*P* < 0.05). In the ZY group, *Dipodascaceae, Metschnikowia_chrysoperlae*, and *g_unidentified* were found only after transportation, whereas *Acremonium, Coprinopsis_cinerea, Cladosporium_sphaerosporum*, and *s_Exophiala* were found only before transportation (*P* < 0.05). After a certain amount of time, the microbiota *Dipodascus* and *Candida_tropicalis* emerged, but the fungi *Alternaria, Pleosporaceae, Sporidiobolaceae* and *Nectriaceae* vanished (*P* < 0.05).

### 3.4. Correlation analysis between microorganism and temperature, nutritional characteristics, as well mycotoxins of WPCS

**Figure 6:**
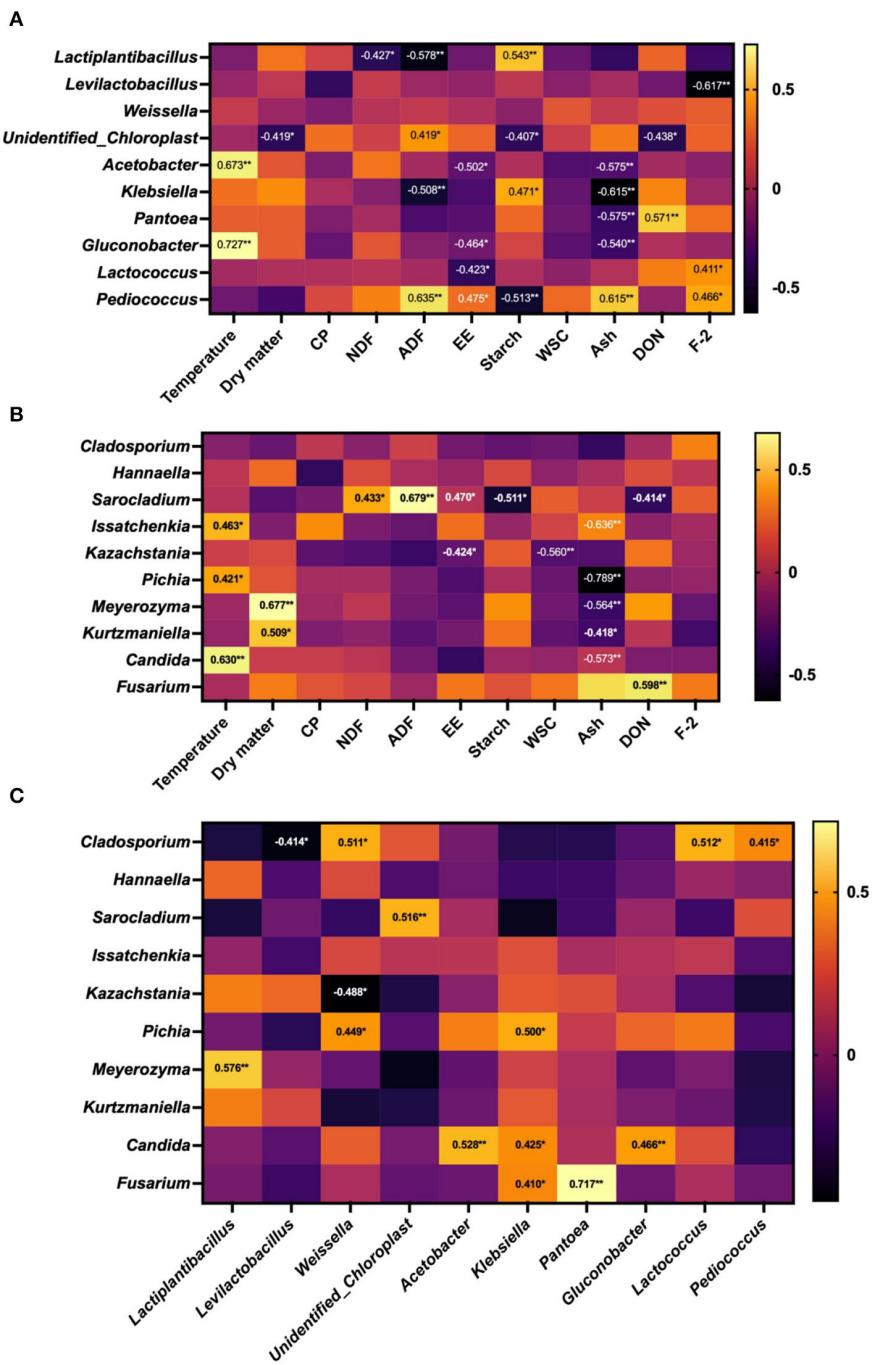
Correlation between microorganism and phenotypic indicators of whole-plant corn silage (WPCS). **(A)** Correlation between the top 10 relatively abundant bacterial genera and temperature, nutrient composition, and mycotoxins in WPCS. **(B)** Correlation between the top 10 relatively abundant fungal genera and temperature, nutrient composition, and mycotoxins in WPCS. **(C)** Correlation between fungal (top 10) and bacterial genera (top 10) in WPCS. ^*^P < 0.05, ^**^P < 0.01.

The correlation between the relative abundance of microbiota and phenotypic indices is shown in Figure 6. The WPCS temperature was positively correlated with the relative abundance of *Acetobacter* and *Glucobacillus* (bacteria), in conjunction with *Issatchenkia, Pichia*, and *Candida* (fungi) (*P* < 0.05). For the relationship between microbiota and nutrients, DM content was negatively correlated with the relative abundance of *unidentified_Chloroplasts* (bacteria) but positively correlated with *Meyerozyma* and *Kazakhstania* (fungi) (*P* < 0.05); the concentration of NDF showed a negative correlation with *Lactobacillus* and a positive correlation with *Sarocladium* (*P* < 0.05); ADF and starch content were correlated with *Lactobacillus, unidentified Chloroplasts, Klebsiella, Pediococcus* (bacteria), in conjunction with *Sarocladium* (fungi) (*P* < 0.05); WCS was only negatively related to *Kazakhstania* (fungi), but the concentration of Ash was affected by both bacteria and fungi abundance. In addition, DON content was influenced by bacteria and fungi, but the concentration of F-2 was only associated with bacteria (*P* > 0.05).

The interaction between bacterial and fungal microorganisms (top 10 relative abundances) of WPCS was also investigated. The strongest positive correlation was observed between *Pantoea* bacteria and *Fusarium* fungi (*P* < 0.01, *r* = 0.717), followed by *Lactiplantibacillus* bacteria and *Meyerozyma* fungi (*P* < 0.01, *r* = 0.576), *Acetobacter* bacteria and *Candida* fungi (*P* < 0.01, *r* = 0.528), *undentified_Chloroplast* bacteria and *Sarocladium* fungi (*P* < 0.01, *r* = 0.516), and *Weissella* and *Lactococcus* bacteria with *Cladosporium* fungi (*P* < 0.05, *r* = 0.511 and *P* < 0.05, *r* = 0.512, respectively). A negative correlation was found between *Levilactobacillus* bacteria and *Cladosporium* fungi (*P* < 0.05, *r* = −0.414), and between *Weissella* bacteria and *Kazachstania* fungi (*P* < 0.05, *R* = −0.488).

## 4. Discussion

### 4.1. Effects of transport time on temperature, nutrients, and mycotoxin content of WPCS

The temperature of corn silage packaged to be sealed immediately after harvest should not exceed 5– 10°C above ambient temperature (27). In the current study, the sample temperatures markedly increased in the ZY and CY groups. This might be because an anaerobic environment formed initially in the middle and bottom of the silage with reduced oxygen content; when the transportation time reached 300 min, lactic acid bacteria began to multiply and decompose the carbohydrates, which further contributed to the increase in temperature (28). Wang et al. (29) suggested that the higher compactness and reduced pore space at the initial stage of fermentation would effectively decrease the corn silage temperature. Therefore, silage should be compacted as much as possible before transportation, which can effectively prevent the loss of nutrients caused by increased temperature (30).

No significant change was found in the contents of DM, ash, NDF, and ADF in WPCS in any group in this study. The content of NDF in silage would generally decrease under fermentation conditions (31). These different results may be due to incomplete anaerobic conditions during transportation. Compared to starch, the fiber is more difficult to utilize by microorganisms under insufficient dissolution time. However, the concentration of starch was significantly reduced when the transport time exceeded 600 min. Starch is a vital indicator of the ensilaging process. As the main substrate source, carbohydrates can be metabolized in the aerobic stage of silage (32), which may explain this result.

The content of mycotoxins is affected by many factors, including oxidation reactions, nutritional conditions, and the environment during the silage fermentation process (33, 34). In the present study, the contents of DON and F-2 in WPCS decreased significantly during short transportation times (< 200 min). This may be due to the loss of corn juice, which contains mycotoxins, in the early stage of transportation. A previous study suggested that excessive juice loss and increased water solubility of mycotoxins are the main reasons for the reduction in mycotoxin content in silage (35). In addition, Richard et al. suggested that the optimum temperature for the formation of F-2 was 28°C, and the improved F-2 content was the result of toxin formation under favorable conditions (9). Thus, the temperature in the present study may be another contributor to the reduction in F-2 content in the DY group.

### 4.2. Effects of transport time on microbial diversity and composition of WPCS

LAB are the major microorganisms involved in silage fermentation, with high activity. Carbohydrates are used to synthesize lactic acid during fermentation, which reduces the pH value and inhibits spoilage microorganisms to ensure excellent silage quality (36). In the present study, as one of the dominant bacterial genera (relative abundance 21.85% - 48.95%) in corn silage, the relative abundance of *Lactiplantibacillus* decreased significantly after transportation for over 300 min, which was consistent with changes in the temperature and starch content. It was speculated that, although microbial communities, mainly including *Lactobacillus, Lactococcus*, and *Streptococcus*, existed on the plant surface before harvest (37), carbohydrates could not be utilized effectively by LAB without an anaerobic environment. Silage in a non-strict anaerobic environment may also attribute to the reproduction of other anaerobic and aerobic bacteria; however, high-throughput sequencing technology could detect only the relative abundance of LAB rather than their specific quantity. In addition, the relative abundance of four bacterial genera, *Pantobacter, Glucococcus, Acetobacter*, and *Acinetobacter*, increased significantly after transportation for more than 300 min. As conditional aerobic or facultative anaerobic pathogens, both *Pantobacter* and *Acinetobacter* exist widely in water and soil and proliferate more easily in moist environments (38). As for the specific reasons why medium and long transport time did not affect mycotoxins and why continuous proliferation of *Pantobacter* and *Acinetobacter* was observed in only ZY, it remains unclear and needs further study in the future works. *Glucobacillus* and *Acetobacter* are acetic acid bacteria that consume residual water-soluble carbohydrates due to insufficient sealing. Wilkinson et al. (39) suggested that lactic acid and acetic acid are oxidized to produce carbon dioxide and water, resulting in the decay and deterioration of fermented feed due to oxygen consumption.

Corn silage is vulnerable to contamination by fungi that distribute air, soil, and environmental dust during harvesting, shredding, or transportation (40). Several toxins caused by fungi are produced during silage fermentation, giving rise to poor quality and safety risks (41). However, unlike bacteria, the fungal diversity of WPCS was not influenced by the transportation time in current study. The result might indicate that the experimental transportation duration was too short to induce the variation of fungal diversity, although the longest time has exceeded 600 min.

### 4.3. Relationship between microorganism and phenotypic indicators of WPCS

Bacteria can secrete enzymes that degrade mycotoxins into non-toxic or less toxic small-molecule metabolites (42). Weinberg et al. (43) found that as silage mycotoxin-degrading bacteria, LAB can be used to improve the safety of silage, and Einezami et al. (44) also reported that LAB and propionic acid bacteria can remove aflatoxins and single-ended sporotoxins from liquid media. In accordance with previous research, a close relationship between microbiota and mycotoxins was found in our study.

DON, produced by *Fusarium* fungi, is widely found in all cereal crops (45). Although correlation analysis showed that the DON content in silage was positively associated with the relative abundance of *Fusarium*, the decreased mycotoxin content in the present study may not be strongly related to the change in fungal abundance. A previous study found that the mycotoxin content and fermentation quality of corn silage were not influenced by the addition of mycotoxin-producing fungi (46). In agreement with this, no relationship between F-2 and *Fusarium* was observed in the present study, although F-2 was produced by *Fusarium*; moreover, the F-2 content was not correlated with other dominant fungi, which may indicate that the competition between bacteria and mycotoxin-producing fungi was a reasonable explanation for the result.

At present, microbial degradation of mycotoxins in silage is a potential and effective technology, mainly through the following two mechanisms: (1) bacterial cells and their supernatant degrade mycotoxins (44); (2) microorganisms (such as LAB yeasts) inhibit mycotoxin production by reducing silage pH, chelation between bacteria and mycotoxins, or competition with mycotoxin-producing fungi (43). Lavermicocca et al. (47) reported that the 10-fold concentrated filtrate of *Lactobacillus plantarum 21b* exhibited high antifungal activity against *Penicillium* spp., *Aspergillus niger, A. flavus* and *Fusarium graminearum*. Hassan et al. (48) attributed antifungal activity to the decreased pH value caused by the production of organic acids during fermentation, which in turn inhibited mold growth. Magnusson and Schnurer (49) found that *Lactobacillus coryneticus Si3* isolated from silage inhibited the growth of *Aspergillus fumigatus, Penicillium Loudi, Fusarium graminearum*, and *F. oxysporum*. Above all, although the specific mechanism of interaction between bacteria and fungi is not clear at present, the development of macrogenomics, macrotranscriptome, and metabolome technology provides novel and effective tools to reveal it in the future.

## 5. Conclusions

In conclusion, the temperature of WPCS increases with rising transport time. The starch content significantly decreased when the transportation time was more than 600 min, which might be associated with a decrease of LAB abundance. Additionally, the mycotoxin variation might be induced by interactions between bacteria and fungi.

## Data availability statement

The datasets presented in this study can be found in online repositories. The names of the repository/repositories and accession number(s) can be found in the article/supplementary material.

## Author contributions

CZ and HW contributed to conception and design of the study. JJ, JL, XZ, and JZ organized the database. JJ and JL performed the statistical analysis. CZ wrote the first draft of the manuscript. CZ, JJ, JL, JZ, and HW wrote sections of the manuscript. All authors contributed to manuscript revision, read, and approved the submitted version.
